# P-1106. Prevalence and disease burden of human cytomegalovirus infection and disease in kidney transplant patients in Taiwan

**DOI:** 10.1093/ofid/ofaf695.1301

**Published:** 2026-01-11

**Authors:** Hsin-Yun Sun, Kai-Ting Chen

**Affiliations:** National Taiwan University Hospital, Taipei, Taipei, Taiwan (Republic of China); MSD Taiwan, Taipei, Taipei, Taiwan (Republic of China)

## Abstract

**Background:**

The human cytomegalovirus (CMV) is a common opportunistic pathogen causing complications in kidney transplant (KT) patients and is associated with poor clinical outcomes. This study assessed the disease burden of CMV infection and disease post-KT in Taiwan.

**Methods:**

This non-interventional, retrospective chart review study assessed medical records on KT patients (aged ≥ 20 years) from the National Taiwan University Hospital Integrated Medical Database. Baseline information 1-year prior and up to 2 years post-KT follow-up data (standard of care, complications, healthcare resource utilization) from patients with donor (D)/recipient (R) CMV serostatus D+/R-, D-/R+, or D+/R+ and had their 1st KT between January 2017-July 2022 were analyzed.

**Results:**

A total of 150 patients (D+/R-: 10.7%; D-/R+: 6.7%; D+/R+: 82.7%) were enrolled. The mean age was 50 years and 62.0% were males. The proportion of CMV DNAemia and disease during the 2-year follow-up was 3.3% (n=5) (D+/R-: 6.3%; D-/R+: 0%; D+/R+: 3.2%) and were treated with valganciclovir (n=3) or tazocin (n=1). D+/R- patients were more likely to have leukopenia (37.5%) and neutropenia (12.5%), with the events also observed in D-/R+ (leukopenia: 0%; neutropenia: 10.0%) and D+/R+ (10.5%; 2.4%) patients. Majority used prophylactic valganciclovir (98.4-100%) for a mean duration of 93-185 days. Among patients, 6.3% D+/R- and 2.4% D+/R+ received hyperimmune globulin for CMV therapy, and 1.6% D+/R+ received G-CSF regimen filgrastim. Patients with CMV disease or any post-KT events had higher 30-day readmission rates and medical utilization due to readmission (hospitalization, costs) and outpatient (visits, costs). Patients with only neutropenia had higher 30-day admission rates and frequent outpatient visits.

**Conclusion:**

Post-KT CMV infection/disease, neutropenia and leukopenia events were observed during the 2-year follow-up, with higher incidence rates seen in D+/R-. The study showed that R+ patients still have a risk of CMV reactivation. A universal prophylaxis for all patients, including R+ patients, is crucial to refine CMV management and prevention to reduce post-KT disease burden.

**Disclosures:**

Kai-Ting Chen, PhD, MSD Taiwan: Employment

